# Import of multidrug-resistant bacteria from abroad through interhospital transfers, Finland, 2010–2019

**DOI:** 10.2807/1560-7917.ES.2021.26.39.2001360

**Published:** 2021-09-30

**Authors:** Mikael Kajova, Tamim Khawaja, Jonas Kangas, Hilda Mäkinen, Anu Kantele

**Affiliations:** 1 Inflammation Center, Department of Infectious Diseases, University of Helsinki and Helsinki University Hospital, Helsinki, Finland; 2 Meilahti Infectious Diseases and Vaccine Research Center, MeiVac, University of Helsinki and Helsinki University Hospital, Helsinki, Finland; 3 Human Microbiome Research Program, Faculty of Medicine, University of Helsinki, Helsinki, Finland

**Keywords:** MDR bacteria, ESBL, hospital transfer

## Abstract

**Background:**

While 20–80% of regular visitors to (sub)tropical regions become colonised by extended-spectrum β-lactamase-producing *Enterobacteriaceae* (ESBL-PE), those hospitalised abroad often also carry other multidrug-resistant (MDR) bacteria on return; the rates are presumed to be highest for interhospital transfers.

**Aim:**

This observational study assessed MDR bacterial colonisation among patients transferred directly from hospitals abroad to Helsinki University Hospital. We investigated predisposing factors, clinical infections and associated fatalities.

**Methods:**

Data were derived from screening and from diagnostic samples collected between 2010 and 2019. Risk factors of colonisation were identified by multivariable analysis. Microbiologically verified symptomatic infections and infection-related mortality were recorded during post-transfer hospitalisation.

**Results:**

Colonisation rates proved highest for transfers from Asia (69/96; 71.9%) and lowest for those within Europe (99/524; 18.9%). Of all 698 patients, 208 (29.8%) were colonised; among those, 163 (78.4%) carried ESBL-PE, 28 (13.5%) MDR *Acinetobacter* species, 25 (12.0%) meticillin-resistant *Staphylococcus aureus*, 25 (12.0%) vancomycin-resistant *Enterococcus*, 14 (6.7%) carbapenemase-producing *Enterobacteriaceae*, and 12 (5.8%) MDR *Pseudomonas aeruginosa*; 46 strains tested carbapenemase gene-positive. In multivariable analysis, geographical region, intensive care unit (ICU) treatment and antibiotic use abroad proved to be risk factors for colonisation. Clinical MDR infections, two of them fatal (1.0%), were recorded for 22 of 208 (10.6%) MDR carriers.

**Conclusions:**

Colonisation by MDR bacteria was common among patients transferred from foreign hospitals. Region of hospitalisation, ICU treatment and antibiotic use were identified as predisposing factors. Within 30 days after transfer, MDR colonisation manifested as clinical infection in more than 10% of the carriers.

## Introduction

The spread of antimicrobial resistance (AMR) is strongly associated with international travel: 20–80% of visitors to high-risk regions become colonised and carry multidrug-resistant (MDR) bacteria back to their home country [1]. In high-income countries, rising background resistance and, particularly, import of MDR bacteria into hospitals from overseas is a concern.

Compared with infections by bacteria susceptible to antibiotics, infections by resistant bacteria are associated with greater mortality, longer hospitalisation and higher costs [2,3]. Colonisation by MDR bacteria, such as extended-spectrum β-lactamase-producing *Enterobacteriaceae* (ESBL-PE), carbapenemase-producing *Enterobacteriaceae* (CPE), meticillin-resistant *Staphylococcus aureus* (MRSA), MDR *Acinetobacter* species (MDRACI), MDR *Pseudomonas aeruginosa* (MDRPA) and vancomycin-resistant *Enterococcus* (VRE), often remains asymptomatic but increases the risk of developing an infection. Colonised individuals may spread bacteria to contacts and the broader environment. Recently, we showed that approximately half of all ESBL-PE imported by travellers carry either intestinal or extraintestinal/uropathogenic virulence genes [4].

Travellers hospitalised abroad are at increased risk of acquiring MDR bacteria [5-13]. In a study by Khawaja et al. in which 1,122 such patients were screened within 12 months after hospitalisation, 55% of those returning from (sub)tropical areas and 17% of those returning from temperate zones carried at least one type of MDR bacteria, mostly ESBL-PE [13].

Research into types and rates of travel-acquired MDR bacteria and the associated risk factors aids prioritisation of infection control resources and selection of empirical antibiotics. Interhospital patient transfers from abroad involve an increased risk of MDR bacterial carriage [13]. Such transfers may pose a substantial threat especially to hospitals in countries with a lower AMR prevalence, yet data on actual rates and risk have thus far been scarce. This study was undertaken to provide region-specific rates intended to provide basis for infection control management when devising guidelines and targeting resources.

## Methods

### Study design

The prevalence and risk factors of colonisation by MDR bacteria were studied among patients transferred from hospitals abroad to Helsinki University Hospital, Finland (HUH) between 1 January 2010 and 30 June 2019. We searched the HUH electronic infection control database for patients screened for both MRSA and MDR Gram-negative bacteria (MDRGNB). The latter screen comprises detection of CPE, ESBL-PE, MDRACI and MDRPA. For such patients, electronic medical records were explored and those with interhospital transfer data included. Patients with records from 2010 to 2013 were covered in a previous AMR investigation [13] that did not report separately on direct hospital transfers.

### Definitions

Hospitalisation abroad was defined as a hospital stay of more than 24 h or an admission involving surgery or some other major invasive procedure. Direct transfer was defined as hospitalisation that continued immediately on return to Finland, with no overnight stay outside hospitals (excluding flights).

### Inclusion criteria

The microbiological inclusion criteria comprised (i) a record of rectal swab or stool sample for MDRGNB screening within 3 days and (ii) MRSA screening from all three sites (nose, throat/trachea, groin/perineum) within 30 days after transfer day. According to HUH guidelines, MDRGNB, MRSA and VRE screening samples are to be taken from all patients transferred directly from hospitals abroad. However, before 2016, this only applied to countries outside the Nordic region. The guidelines also advise sampling of skin lesions, urine from in-dwelling catheters in place for over 7 days, and trachea from intubated/tracheostomy patients. During the study period from 2010 to 2019, some (mostly minor) modifications were made to the guidelines, summarised in the Supplement (Supplementary Table S1). Two or three specimens were recommended for each item/area on separate days. A VRE screening was not included as a criterion since it had not been performed regularly, but VRE results were recorded when available. We recorded all findings of MDR bacteria in screening and diagnostic samples collected within 30 days from the first screening.

### Exclusion criteria

Medical records were searched for other documented stays and hospitalisations abroad. Stays outside Europe in regions other than those where hospitalised during the previous 12 months led to exclusion. Since travel within Europe is common and often not recorded in patient files, this was not taken as an exclusion criterion. Patients with multiple foreign hospitalisations were included only if all the hospitalisations took place in the same European country or, for other parts of the world, within the same geographical region.

### Data collection

We collected data on underlying diseases, chronic alcohol abuse, travel-related factors and information linked to hospitalisation (antibiotics, interventions, intensive care unit (ICU) treatment, duration, diagnosis), and calculated the Charlson comorbidity index (CCI) [14]. In addition to documented antibiotic use, records of bacterial infections known to require antibiotics or surgery for which prophylaxis is advised were classified as ‘antibiotic use abroad’. Ongoing antibiotic treatments at the time of screening were recorded but marked as negative if at least one set of samples had been taken without any antibiotics administered for 24 h. Because of the complexity of potential antimicrobial effects, we chose not to consider whether or not an antibiotic acted against the MDR bacteria carried by the patient.

Symptomatic, microbiologically verified MDR infections and direct mortality caused by such infections were assessed by an infectious diseases specialist; they were recorded from transfer until discharge or for a maximum of 30 days. Because of incomplete data, potential MDR bacterial infections treated abroad were not evaluated.

### Microbiological methods

The various MDR bacteria were identified by the standard methods used in the HUH laboratory HUSLAB, as follows.

 MRSA was identified after overnight enrichment on eMRSA broth (Copan Italia, Brescia, Italy) or selective in-house MRSA enrichment broth [15] and subsequent culture on chromID MRSA (bioMérieux, Marcy-l’Étoile, France) or CHROMagar MRSA (CHROMagar, Paris, France), and confirmed by quantitative PCR for the *S. aureus*-specific nuclease and *mecA* gene [15].

VRE were detected using enrichment Enterococcosel broth (BBL, Cockeysville, MD) followed by culture on in-house selective media, as previously described [15], or CHROMagar VRE media. Positive findings were confirmed by in-house PCR [15].

ESBL-PE and CPE were analysed by plating directly on CHROMagar ESBL and CHROMagar *Klebsiella pneumoniae* Carbapenemase (KPC) or CHROMagar mSuperCARBA, respectively. Identification of ESBL-PE species was confirmed by matrix-assisted laser desorption ionisation time-of-flight (MALDI-TOF; Vitek-MS, bioMérieux) and resistance was determined according to the guidelines from the Clinical and Laboratory Standards Institute (CLSI) and, from 2011, the European Committee on Antimicrobial Susceptibility testing (EUCAST) [15-17]. CPE were confirmed with in-house PCR targeting the carbapenemase gene [15].

MDRACI and MDRPA were screened on ESBL and KPC plates. Cultures were tested by C-390, VITEK-GN or MALDI-TOF for species identification. *Acinetobacter* isolates resistant to meropenem and *Pseudomonas* isolates resistant to both meropenem and ceftazidime were analysed by PCR for carbapenemase genes [15].

### Statistical analyses

We used SPSS v. 25.0 (IBM Corp., Armonk, New York, United States) for all statistical analyses. For univariate analyses the chi-squared test, Fisher’s exact test or binary logistic regression were used, as appropriate. For multivariable analysis, variables with a p value below 0.2 in univariate analysis, or those assessed as clinically relevant, were included. In cases of two strongly correlating explanatory variables, only one was chosen. The most parsimonious model was found by backward selection based on Akaike information criteria.

### Ethical statement

The present study was approved by the research board of HUH Department of Internal Medicine. Since this investigation did not involve an intervention, an ethics committee review was not required (Finnish Medical Research Act).

## Results

### Study population

**Figure 1:**
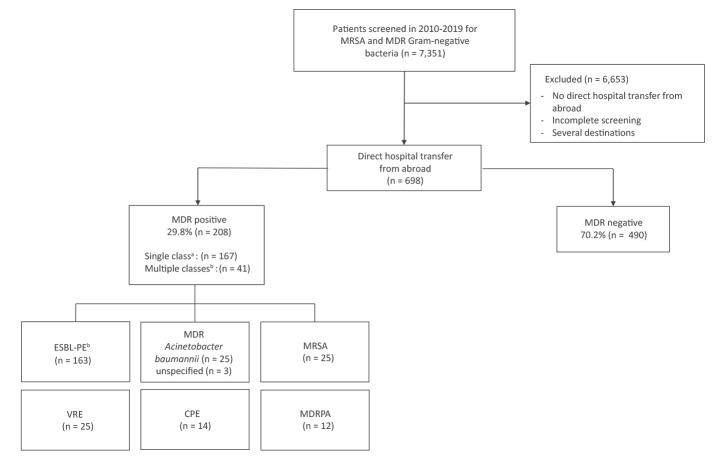
Multidrug-resistant bacterial colonisation among patients transferred directly from hospitals abroad to Helsinki University Hospital, Finland, January 2010– June 2019 (n = 698)

CPE: carbapenemase-producing *Enterobacteriaceae*; ESBL-PE: extended-spectrum β-lactamase-producing *Enterobacteriaceae*; MDR: multidrug-resistant; MDRPA: multidrug-resistant *Pseudomonas aeruginosa*; MRSA: meticillin-resistant *Staphylococcus aureus*; VRE: vancomycin-resistant *Enterococcus.*

^a^ MDR bacterial classes: CPE, ESBL-PE, MDR *Acinetobacter* species, MDRPA, MRSA, VRE. A patient denoted with single class had one or more isolates of the same MDR bacterial class, one denoted with multiple classes had isolates of two or more MDR bacterial classes.

^b^ In addition, 10 patients carried non-ESBL *Enterobacteriaceae* resistant to third-generation cephalosporins.

A total of 698 patients undergoing direct hospital transfers between 1 January 2010 and 30 June 2019 met the inclusion criteria (Figure 1). Of the 86 countries where initial hospitalisation occurred, Spain, Estonia and Thailand accounted for 310 patients (44.4%). Europe was the most common region with 524 patients (75.1%). The majority of those returning from Asia (69/96; 71.9%) had been hospitalised in South East Asia. For a list of the countries in which hospitalisation took place, see Supplement (Supplementary Table S2). Residence abroad was recorded as type of travel for 96 patients (13.8%), visiting friends and relatives (VFR) for 49 (7.0%), and work, holiday and other reasons for the rest (553; 79.2%).

The median duration of hospitalisation abroad was 9 days (range: 1–643). A total of 190 (27.2%) patients had been admitted to ICU and 290 (41.5%) had undergone surgery abroad. The primary diagnosis on repatriation was non-trauma disease for 375 (53.7%) patients and trauma for 323 (46.3%) patients.

### Antibiotic use abroad and during screening

The medical records included documentation of antibiotic use abroad for 383 patients (54.9%). After transfer, 458 (65.6%) patients had three-site MRSA and faecal MDRGNB screening at least once without ongoing antibiotic treatment, while 182 (26.1%) had ongoing antibiotic therapy at screenings. For 58 patients, data were missing or antibiotics were used during part of the screenings.

### Findings of multidrug-resistant bacteria

**Figure 2:**
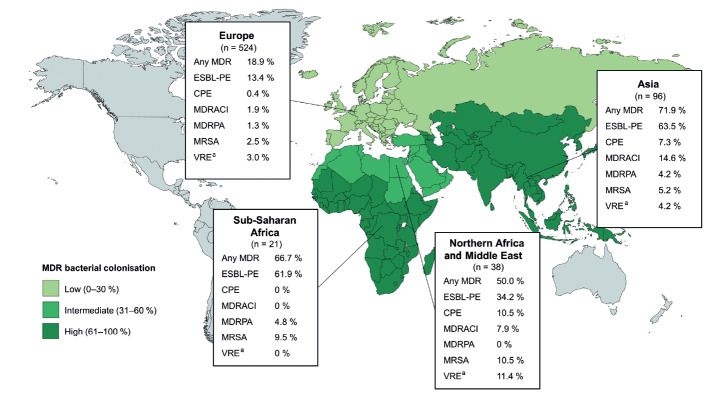
Colonisation by multidrug-resistant bacteria of patients transferred directly from hospitals abroad to Helsinki University Hospital, Finland, 2010–2019 (n = 698)

CPE: carbapenemase-producing *Enterobacteriaceae*; ESBL-PE: extended-spectrum β-lactamase-producing *Enterobacteriaceae*; MDR: multidrug-resistant; MDRACI: multidrug-resistant *Acinetobacter*; MDRPA: multidrug-resistant *Pseudomonas aeruginosa*; MRSA: meticillin-resistant *Staphylococcus aureus*; VRE: vancomycin-resistant *Enterococcus*.

A total of 208 patients (29.8%) were colonised by MDR bacteria, 41 by more than one class of MDR bacteria (Figure 1). ESBL-PE were the most common findings with 163 (23.4%) carriers, 76 of them (10.9% of the total study population) showing solely ESBL-producing *Escherichia coli.* Twenty-eight patients carried MDRACI, 25 carried MRSA, 14 carried CPE and 12 MDRPA, while 25 had VRE; data on VRE screening were missing for 35. Comparisons between the various years did not reveal significant differences in total MDR bacterial carriage rates 2010–2019 (Supplement, Supplementary Table S3). Figure 2 shows the colonisation rates by geographical regions.

Other continents are not shown because of low number of cases: Latin America and the Caribbean (n = 10), Northern America (n = 3), Oceania (n = 6). The government-controlled area of Cyprus is included under Europe.

^a^ VRE % given of screened patients.

The map was created with mapchart.net.

Culture-based analyses identified 261 ESBL-PE, 25 MRSA, 26 VRE, 14 MDRPA, 24 CPE and 33 MDRACI strains, 30 of which were confirmed as *Acinetobacter baumannii* and three as non-specified *Acinetobacter* species. Among ESBL-PE carriers, for 102 patients (62.6%) one ESBL-PE strain had been recorded, for 33 (20.2%) two, and for 28 (17.2%) three or more. A total of 46 carbapenemase gene-positive (CPE, MDRACI or MDRPA) strains had been recorded for 33 patients, seven carbapenemase gene-negative MDRPA strains for six and one gene-negative MDRACI strain for one. From 2010 to 2012, carbapenemase genes were not tested in five MDRPA strains carried by four patients, nor in four MDRACI strains, each from one patient. The carbapenemase gene-negative and non-tested strains were included in the figures for colonised patients.

### Risk factors for colonisation with multidrug-resistant bacteria

In univariate analysis, several factors were associated with MDR carriage (Table 1) and in multivariable analysis, geographical region, ICU treatment and antibiotic use abroad were identified as independent risk factors. Separate analyses of individual MDR types are presented below.

**Table 1:** Patient characteristics and risk factor analyses of multidrug-resistant bacterial colonisation among patients screened after direct transfer from hospitals abroad to Helsinki University Hospital, Finland, 2010–2019 (n = 698)

	Patients (n = 698)	MDR bacteria positive (n = 208)		MDR bacteria negative (n = 490)		OR (95% CI) in univariate analysis	p value in univariate analysis	AOR (95% CI) in multivariable analysis^a^	p value in multivariable analysis^a^
	n	n	%	n	%				
Sex									
Male	427	134	31.4	293	68.6	1.2 (0.9–1.7)	0.25	NI	NI
Female	271	74	27.3	197	72.7	Ref	Ref	NI	NI
Age groups (years)							0.55	NA	NA
0–5	27	4	14.8	23	85.2	Ref	Ref	NI	NI
6–17	35	9	25.7	26	74.3	2.0 (0.5–7.3)	0.30	NI	NI
18–30	61	19	31.1	42	68.9	2.6 (0.8–8.6)	0.12	NI	NI
31–50	145	43	29.7	102	70.3	2.4 (0.8–7.4)	0.12	NI	NI
51–65	175	58	33.1	117	66.9	2.9 (0.9–8.6)	0.06	NI	NI
> 65	255	75	29.4	180	70.6	2.4 (0.8–7.2)	0.12	NI	NI
CCI							0.61	NA	NA
0–1 points	428	130	30.4	298	69.6	Ref	Ref	NI	NI
2–4 points	217	60	27.6	157	72.4	0.9 (0.6–1.3)	0.47	NI	NI
> 4 points	53	18	34.0	35	66.0	1.2 (0.6–2.2)	0.59	NI	NI
Chronic alcohol abuse									
Yes	68	22	32.4	46	67.6	1.1 (0.7–2.0)	0.63	NI	NI
No	630	186	29.5	444	70.5	Ref	Ref	NI	NI
Travel type							0.07	Eliminated^b^	Eliminated^b^
Work/leisure/other	553	154	27.8	399	72.2	Ref	Ref	NI	NI
Residence abroad	96	34	35.4	62	64.6	1.4 (0.9–2.2)	0.13	NI	NI
VFR	49	20	40.8	29	59.2	1.8 (1.0–3.3)	0.06	NI	NI
Geographical region							< 0.01	NA	< 0.01
North America	3	1	33.3	2	66.7	2.1 (0.2–23.9)	0.54	1.4 (0.1–16.0)	0.79
Latin America, Caribbean	10	6	60.0	4	40.0	6.4 (1.8–23.3)	< 0.01	6.4 (1.7–23.7)	0.01
Sub-Saharan Africa	21	14	66.7	7	33.3	8.6 (3.4–21.8)	< 0.01	9.8 (3.8–25.4)	< 0.01
North Africa, Middle East	38	19	50.0	19	50.0	4.3 (2.2–8.4)	< 0.01	4.1 (2.1–8.2)	< 0.01
Asia	96	69	71.9	27	28.1	11.0 (6.7–18.0)	< 0.01	10.5 (6.3–17.3)	< 0.01
Oceania	6	0	0	6	100	NA	NA	NA	NA
Europe	524	99	18.9	425	81.1	Ref	Ref	Ref	Ref
Duration of hospitalisation							< 0.01	Eliminated^b^	Eliminated^b^
1–2 days	83	14	16.9	69	83.1	Ref	Ref	NI	NI
3–7 days	208	48	23.1	160	76.9	1.5 (0.8–2.9)	0.25	NI	NI
8–14 days	186	56	30.1	130	69.9	2.1 (1.1–4.1)	0.02	NI	NI
Over 14 days	193	77	39.9	116	60.1	3.3 (1.7–6.2)	< 0.01	NI	NI
Data missing	28	13	46.4	15	53.6	NI	NI	NI	NI
ICU treatment abroad									
Yes	190	76	40.0	114	60.0	1.9 (1.3–2.7)	< 0.01	1.8 (1.2–2.7)	0.01
No	508	132	26.0	376	74.0	Ref	Ref	Ref	Ref
Major invasive procedure abroad								Eliminated^b^	Eliminated^b^
Yes	290	102	35.2	188	64.8	1.5 (1.1–2.1)	0.01	NI	NI
No	408	106	26.0	302	74.0	Ref	Ref	NI	NI
Antibiotic use abroad									
Yes	383	141	36.8	242	63.2	2.2 (1.5–3.0)	< 0.01	1.6 (1.1–2.4)	0.02
No	315	67	21.3	248	78.7	Ref	Ref	Ref	Ref
Reason for hospitalisation									
Trauma	323	96	29.7	227	70.3	Ref	Ref	NI	NI
Non-trauma	375	112	29.9	263	70.1	1.0 (0.7–1.4)	0.97	NI	NI
Antibiotic use during screening									
Yes	182	69	37.9	113	62.1	1.8 (1.2–2.6)	< 0.01	NI	NI
No	458	117	25.5	341	74.5	Ref	Ref	NI	NI
Data missing	58	22	37.9	36	62.1	NI	NI	NI	NI

AOR: adjusted odds ratio; CCI: Charlson Comorbidity Index; CI: confidence interval; ICU: intensive care unit; NA: not applicable; NI: not included; OR: odds ratio; Ref: reference; VFR: visiting friends and relatives.

^a^ The following variables were included in multivariable analysis: travel type, geographical region, duration of hospitalisation, ICU treatment, major invasive procedure, antibiotic use abroad.

^b^ Eliminated before final step in backward selection.

Univariate analysis indicated that country of hospitalisation, antibiotic use abroad, duration of hospitalisation and surgery were risk factors associated with ESBL-PE carriage (Table 2), whereas multivariable analysis yielded only geographical region as risk factor.

**Table 2:** Patient characteristics and risk factor analyses of ESBL-producing *Enterobacteriaceae* colonisation among patients screened after direct transfer from hospitals abroad to Helsinki University Hospital, Finland, 2010–2019 (n = 698)

	Patients (n = 698)	ESBL-PE-positive (n = 163)		ESBL-PE-negative (n = 535)		OR (95% CI) in univariate analysis	p value in univariate analysis	AOR (95% CI) in multivariable analysis^a^	p value in multivariable analysis^a^
	n	n	%	n	%				
Sex									
Male	427	105	24.6	322	75.4	1.2 (0.8–1.7)	0.33	NI	NI
Female	271	58	21.4	213	78.6	Ref	Ref	NI	NI
Age group (years)							0.39	NA	
0–5	27	4	14.8	23	85.2	Ref	Ref	NI	NI
6–17	35	9	25.7	26	74.3	2.0 (0.5–7.3)	0.30	NI	NI
18–30	61	16	26.2	45	73.8	2.0 (0.6–6.8)	0.25	NI	NI
31–50	145	36	24.8	109	75.2	1.9 (0.6–5.9)	0.27	NI	NI
51–65	175	48	27.4	127	72.6	2.2 (0.7–6.6)	0.17	NI	NI
> 65	255	50	19.6	205	80.4	1.4 (0.5–4.2)	0.55	NI	NI
CCI							0.64	NA	
0–1 points	428	105	24.5	323	75.5	Ref	Ref	NI	NI
2–4 points	217	47	21.7	170	78.3	0.9 (0.6–1.3)	0.42	NI	NI
> 4 points	53	11	20.8	42	79.2	0.8 (0.4–1.6)	0.55	NI	N
Chronic alcohol abuse									
Yes	68	14	20.6	54	79.4	Ref	Ref	NI	NI
No	630	149	23.7	481	76.3	1.2 (0.6–2.2)	0.57	NI	NI
Travel type							0.26	NA	
Work/leisure/other	553	122	22.1	431	77.9	Ref	Ref	NI	NI
Residence abroad	96	26	27.1	70	72.9	1.3 (0.8–2.1)	0.28	NI	NI
VFR	49	15	30.6	34	69.4	1.6 (0.8–3.0)	0.17	NI	NI
Geographical region							< 0.01	NA	< 0.01
North America	3	0	0	3	100	NA	NA	NA	NA
Latin America, Caribbean	10	6	60.0	4	40.0	9.7 (2.7–35.3)	< 0.01	9.7 (2.7–35.4)	< 0.01
Sub-Saharan Africa	21	13	61.9	8	38.1	10.5 (4.2–26.3)	< 0.01	13.1 (4.7–37.0)	< 0.01
North Africa, Middle East	38	13	34.2	25	65.8	3.4 (1.6–6.9)	< 0.01	3.7 (1.8–7.6)	< 0.01
Asia	96	61	63.5	35	36.5	11.3 (7.0–18.4)	< 0.01	10.4 (6.3–17.2)	< 0.01
Oceania	6	0	0	6	100	NA	NA	NA	NA
Europe	524	70	13.4	454	86.6	Ref	Ref	Ref	Ref
Duration of hospitalisation							0.03	Eliminated^b^	Eliminated^b^
1–2 days	83	12	14.5	71	85.5	Ref	Ref	NI	NI
3–7 days	208	39	18.8	169	81.3	1.4 (0.7–2.8)	0.39	NI	NI
8–14 days	186	46	24.7	140	75.3	1.9 (1.0–3.9)	0.06	NI	NI
Over 14 days	193	55	28.5	138	71.5	2.4 (1.2–4.7)	0.01	NI	NI
Data missing	28	11	39.3	17	60.7	NI	NI	NI	NI
ICU treatment abroad									
Yes	190	50	26.3	140	73.7	1.2 (0.9–1.8)	0.26	NI	NI
No	508	113	22.2	395	77.8	Ref	Ref	NI	NI
Major invasive procedure abroad									
Yes	290	79	27.2	211	72.8	1.4 (1.0–2.1)	0.04	1.5 (1.0–2.2)	0.07
No	408	84	20.6	324	79.4	Ref	Ref	Ref	Ref
Antibiotic use abroad								Eliminated^b^	Eliminated^b^
Yes	383	104	27.2	279	72.8	1.6 (1.1–2.3)	0.01	NI	NI
No	315	59	18.7	256	81.3	Ref	Ref	NI	NI
Reason for hospitalisation									
Trauma	323	79	24.5	244	75.5	1.1 (0.8–1.6)	0.52	NI	NI
Non-trauma	375	84	22.4	291	77.6	Ref	Ref	NI	NI
Antibiotic use during screening^c^									
Yes	199	51	25.6	148	74.4	1.3 (0.9–1.9)	0.23	NI	NI
No	472	101	21.4	371	78.6	Ref	Ref	NI	NI
Data missing	27	11	40.7	16	59.3	NI	NI	NI	NI

AOR: adjusted odds ratio; CCI: Charlson Comorbidity Index; CI: confidence interval; ICU: intensive care unit; ESBL-PE: extended-spectrum β-lactamase-producing Enterobacteriaceae; NA: not applicable; NI: not included; OR: odds ratio; Ref: reference; VFR: visiting friends and relatives.

^a^ The following variables were included in multivariable analysis: geographical region, duration of hospitalisation, major invasive procedure, antibiotic use abroad.

^b^ Eliminated before final step in backward selection.

^c^ Antibiotic treatment during screening for multidrug-resistant Gram-negative bacteria (MDRGNB).

Results of the univariate analyses conducted for MDRACI and MRSA are presented in Tables 3 and 4. Carriage of each of these two was associated with antibiotic use abroad, while only MDRACI carriage was associated also with antibiotics during screening. In addition, both were associated with ICU treatment abroad. Colonisation with MDRACI was more common among those hospitalised in Asia (14/96; 14.6%) than Europe (10/524; 1.9%). Carriage of MRSA was associated with chronic alcohol abuse, a finding independent of antibiotic use in a model with two explanatory variables. Significant associations were also recorded between CCI and MDRACI (Table 3) and between duration of hospitalisation and MRSA (Table 4)

**Table 3:** Patient characteristics and risk factor analyses of multidrug-resistant *Acinetobacter* spp. colonisation among patients screened after direct transfer from hospitals abroad to Helsinki University Hospital, Finland, 2010–2019 (n = 698)

	Patients (n = 698)	MDRACI-positive (n = 28)		MDRACI-negative (n = 670)		OR (95% CI) in univariate analysis	p value in univariate analysis
	n	n	%	n	%		
**Sex**							
Male	427	21	4.9	406	95.1	2.0 (0.8–4.7)	0.13
Female	271	7	2.6	264	97.4	Ref	Ref
Age group (years)							0.34
0–5	27	0	0	27	100	NA	NA
6–17	35	1	2.9	34	97.1	0.5 (0.1–3.7)	0.47
18–30	61	2	3.3	59	96.7	0.5 (0.1–2.4)	0.43
31–50	145	8	5.5	137	94.5	0.9 (0.4–2.3)	0.88
51–65	175	2	1.1	173	98.9	0.2 (0.0–0.8)	0.03
> 65	255	15	5.9	240	94.1	1.0	Ref
CCI							0.02
0–1 points	428	10	2.3	418	97.7	Ref	Ref
2–4 points	217	14	6.5	203	93.5	2.9 (1.3–6.6)	0.01
> 4 points	53	4	7.5	49	92.5	3.4 (1.0–11.3)	0.04
Chronic alcohol abuse							
Yes	68	3	4.4	65	95.6	1.1 (0.3–3.8)	0.75
No	630	25	4.0	605	96.0	Ref	Ref
Travel type							0.22
Work/leisure/other	553	19	3.4	534	96.6	Ref	Ref
Residence abroad	96	7	7.3	89	92.7	2.2 (0.9–5.4)	0.08
VFR	49	2	4.1	47	95.9	1.2 (0.3–5.3)	0.81
Geographical region							< 0.01
North America	3	0	0	3	100	NA	NA
Latin America, Caribbean	10	1	10.0	9	90.0	5.7 (0.7–49.5)	0.11
Sub-Saharan Africa	21	0	0	21	100	NA	NA
North Africa, Middle East	38	3	7.9	35	92.1	4.4 (1.2–16.7)	0.03
Asia	96	14	14.6	82	85.4	8.8 (3.8–20.4)	< 0.01
Oceania	6	0	0	6	100	NA	NA
Europe	524	10	1.9	514	98.1	Ref	Ref
Duration of hospitalisation							0.14
1–2 days	83	1	1.2	82	98.8	Ref	Ref
3–7 days	208	6	2.9	202	97.1	2.4 (0.3–20.5)	0.41
8–14 days	186	7	3.8	179	96.2	3.2 (0.4–26.5)	0.28
Over 14 days	193	13	6.7	180	93.3	5.9 (0.8–46.0)	0.09
Data missing	28	1	3.6	27	96.4	NI	NI
ICU treatment abroad							
Yes	190	20	10.5	170	89.5	7.4 (3.2–17.0)	< 0.01
No	508	8	1.6	500	98.4	Ref	Ref
Major invasive procedure abroad							
Yes	290	16	5.5	274	94.5	1.9 (0.9–4.1)	0.09
No	408	12	2.9	396	97.1	Ref	Ref
Antibiotic use abroad							
Yes	383	27	7.0	356	93.0	23.8 (3.2–176.3)	< 0.01
No	315	1	0.3	314	99.7	Ref	Ref
Reason for hospitalisation							
Trauma	323	12	3.7	311	96.3	Ref	Ref
Non-trauma	375	16	4.3	359	95.7	1.2 (0.5–2.5)	0.71
Antibiotic use during screening^a^							
Yes	199	13	6.5	186	93.5	2.9 (1.3–6.7)	< 0.01
No	472	11	2.3	461	97.7	Ref	Ref
Data missing	27	4	14.8	23	85.2	NI	NI

AOR: adjusted odds ratio; CCI: Charlson Comorbidity Index; CI: confidence interval; ICU: intensive care unit; NA: not applicable; NI: not included; OR: odds ratio; Ref: reference; VFR: visiting friends and relatives.

^a^ Antibiotic treatment during screening for multidrug-resistant Gram-negative bacteria (MDRGNB).

**Table 4:** Patient characteristics and risk factor analyses of meticillin-resistant *Staphylococcus aureus* colonisation among patients screened after transfer directly from hospitals abroad to Helsinki University Hospital, Finland, 2010–2019 (n = 698)

	Patients (n = 698)	MRSA positive (n = 25)		MRSA negative (n = 673)		OR (95% CI) in univariate analysis	p value in univariate analysis	AOR (95% CI) in multivariable analysis^a^	p value in multivariable analysis^a^
	n	n	%	n	%				
**Sex**									
Male	427	17	4.0	410	96.0	1.4 (0.6–3.2)	0.48	NI	NI
Female	271	8	3.0	263	97.0	Ref	Ref	NI	NI
Age group (years)							0.69	NA	
0–5	27	0	0	27	100	NA	NA	NI	NI
6–17	35	1	2.9	34	97.1	0.5 (0.1–4.3)	0.57	NI	NI
18–30	61	1	1.6	60	98.4	0.3 (0.0–2.4)	0.26	NI	NI
31–50	145	3	2.1	142	97.9	0.4 (0.1–1.4)	0.15	NI	NI
51–65	175	7	4.0	168	96.0	0.8 (0.3–2.0)	0.60	NI	NI
> 65	255	13	5.1	242	94.9	Ref	Ref	NI	NI
CCI							0.36	NA	
0–1 points	428	12	2.8	416	97.2	Ref	Ref	NI	NI
2–4 points	217	10	4.6	207	95.4	1.7 (0.7–3.9)	0.24	NI	NI
> 4 points	53	3	5.7	50	94.3	2.1 (0.6–7.6)	0.27	NI	NI
Chronic alcohol abuse									
Yes	68	6	8.8	62	91.2	3.1 (1.2–8.1)	0.03	2.9 (1.1–7.5)	0.03
No	630	19	3.0	611	97.0	Ref	Ref	Ref	Ref
Travel type							0.56	NA	
Work/leisure/other	553	18	3.3	535	96.7	Ref	Ref	NI	NI
Residence abroad	96	4	4.2	92	95.8	1.3 (0.4–3.9)	0.65	NI	NI
VFR	49	3	6.1	46	93.9	1.9 (0.6–6.8)	0.30	NI	NI
Geographical region							0.14	NA	
North America	3	0	0	3	100	NA	NA	NI	NI
Latin America, Caribbean	10	1	10.0	9	90.0	4.4 (0.5–37.1)	0.18	NI	NI
Sub-Saharan Africa	21	2	9.5	19	90.5	4.1 (0.9–19.6)	0.07	NI	NI
North Africa, Middle East	38	4	10.5	34	89.5	4.6 (1.4–14.9)	0.01	NI	NI
Asia	96	5	5.2	91	94.8	2.2 (0.8–6.2)	0.15	NI	NI
Oceania	6	0	0	6	100	NA	NA	NI	NI
Europe	524	13	2.5	511	97.5	Ref	Ref	NI	NI
Duration of hospitalisation							0.03	NA	
1–2 days	83	0	0	83	100	NA	NA	NI	NI
3–7 days	208	3	1.4	205	98.6	0.2 (0.0-.0.6)	< 0.01	NI	NI
8–14 days	186	6	3.2	180	96.8	0.4 (0.2–1.0)	0.06	NI	NI
Over 14 days	193	15	7.8	178	92.2	Ref	Ref	NI	NI
Data missing	28	1	3.6	27	96.4	NI	NI	NI	NI
ICU treatment abroad									
Yes	190	12	6.3	178	93.7	2.6 (1.2–5.7)	0.02	NI	NI
No	508	13	2.6	495	97.4	Ref	Ref	NI	NI
Major invasive procedure abroad									
Yes	290	14	4.8	276	95.2	1.8 (0.8–4.1)	0.14	NI	NI
No	408	11	2.7	397	97.3	Ref	Ref	NI	NI
Antibiotic use abroad									
Yes	383	19	5.0	364	95.0	2.7 (1.1–6.8)	0.03	2.5 (1.0–6.5)	0.05
No	315	6	1.9	309	98.1	Ref	Ref	Ref	Ref
Reason for hospitalisation									
Trauma	323	11	3.4	312	96.6	Ref	Ref	NI	NI
Non-trauma	375	14	3.7	361	96.3	1.1 (0.5–2.5)	0.82	NI	NI
Antibiotic use during MRSA screening									
Yes	185	9	4.9	176	95.1	1.7 (0.7–4.0)	0.22	NI	NI
No	477	14	2.9	463	97.1	Ref	Ref	NI	NI
Data missing	36	2	5.6	34	94.4	NI	NI	NI	NI

AOR: adjusted odds ratio; CCI: Charlson Comorbidity Index; CI: confidence interval; ICU: intensive care unit; NA: not applicable; NI: not included; OR: odds ratio; Ref: reference; VFR: visiting friends and relatives.

^a^ Due to a low number of MRSA positive cases (n = 25), only the following variables were included in the multivariable model: chronic alcohol abuse, antibiotic use abroad.

For VRE, the following factors were associated with colonisation: antibiotic use abroad (odds ratio (OR) = 3.4; 95% confidence interval (CI): 1.3–9.2; p = 0.01), antibiotic use during VRE screening (OR = 2.3; 95% CI: 1.0–5.3; p = 0.048), ICU treatment abroad (OR = 3.0; 95% CI: 1.3–6.7; p = 0.005), and travel type (travellers VFR vs work/holiday/other OR = 5.2; 95% CI: 1.9–14.3; p = 0.001). As for other factors in Tables 3 and 4, no significant association was found (data not shown).

Risk factor analyses were not carried out for CPE or MDRPA due to the small number of colonised individuals (14 with CPE and 12 with MDRPA).

### Clinical infections caused by multidrug-resistant bacteria

During post-transfer hospitalisation, 22 of 698 patients (3.2% of the whole study population and 10.6% of those identified as MDR bacteria carriers) had a microbiologically verified clinical MDR bacterial infection, most commonly pneumonia which was found in nine patients (1.3% of all), surgical site infection (six patients, 0.9%), and urinary tract infection (six patients, 0.9%). One patient had MDR bacteraemia. For sites of infection and causative MDR bacteria, see Supplement (Supplementary Table S4). Infection by MDR bacteria was recorded as cause of death for two patients (0.3%).

## Discussion

Of the 698 patients transferred to a Finnish hospital directly from hospitals abroad, 29.8% were colonised by MDR bacteria, the rates varying considerably by geographical region visited. From these 208 patients, 383 MDR bacterial strains were recorded. While these figures indicate the burden of MDR bacteria related to interhospital transfer, closer scrutiny reveals background data applicable to infection control practices and even choice of empiric antibiotics.

At first glance, the 29.8% overall MDR carriage rate appears low. As the rates typically decrease after patients return to low-prevalence countries [18-23], one could expect colonisation to be particularly common in these patients who were screened soon after return to Finland. By contrast, the prevalence proved to be similar to that of our previous data from 2010 to 2013 showing 29.7% carriage rates for patients screened within 12 months after hospitalisation abroad [13]. However, the closeness of the rates may be ascribed to at least two factors. Firstly, up to 23% of the patients in that previous investigation were, in fact, direct-transfer patients, and the time taken from return from abroad to sampling for the rest of the patients was short (median: 11 days). Secondly, the proportion of patients hospitalised in Europe where acquisition is less common [13,15] was higher in the current (75%) than in the previous dataset (64%).

Other European studies have reported MDR bacterial colonisation in similar patient groups but with differences in research design [5-9,11,12]. Among 1,167 patients directly transferred from hospitals abroad to the Netherlands between 1998 and 2001, Kaiser et al. show a colonisation rate of 18.2% [6], but their designation of resistant Gram-negative bacteria was solely based on gentamicin resistance [6] and there has been a substantial general increase in AMR rates since that study. In more recent research among patients screened on direct transfer or within 14 days of hospitalisation abroad, between 7.2 and 28.6% were colonised [7-9,11,12]. For patients hospitalised abroad and examined within 14 days in Switzerland between 2009 and 2011, Nemeth et al. showed the rate to be 17%, although VRE was not included in this study [7]. In another Swiss study where outpatient treatment abroad was also included but without screening for VRE, Kaspar et al. report a 16.3% carriage rate for direct transfer patients in 2012 to 2013 [11]. Josseaume et al. (2010–2011) and Birgand et al. (2012–2013) show MDR bacterial colonisation for 7.2% and 28.6% of repatriated patients in France, respectively [8,9]. In 2012 to 2013, Mutters et al. detected a colonisation rate of 21.0% among patients hospitalised abroad at least 48 h and subsequently transferred to a German hospital [12].

The present study identified region visited, ICU treatment, and antibiotic use during travel as independent risk factors of MDR bacterial colonisation. All three factors have previously been associated with MDR acquisition [13,24-27]. In our study population, association with geographical region proved particularly strong, for example, the colonisation rate was 18.9% among patients transferred from European countries and 71.9% among those returned from Asia (p < 0.001; OR = 10.5; 95% CI: 6.3–17.3). This difference was mainly ascribed to Gram-negative MDR bacteria rather than MRSA or VRE.

The overall colonisation rate by ESBL-PE was 23.4%, accounting for 68.1% of all MDR strains identified. This rate is substantially higher than among the general Finnish population: in 2009 to 2010, 1.2% of 430 Finnish travellers were colonised with faecal ESBL-PE before travel [27]; in 2015 to 2017, a study among Finnish elective surgery patients and medical students reported that 4.7% were colonised with ESBL-producing *E. coli*, and 1.1% with ESBL-producing *K. pneumoniae* [28].

Several prospective investigations have shown that visitors to (sub)tropical regions acquire ESBL-PE, with antibiotic use predisposing to colonisation [18,27,29]. In the present study looking at hospitalised travellers, multivariable analysis identified region of hospitalisation as the sole factor independently associated with ESBL-PE colonisation. ESBL-PE acquisition is so common among travellers visiting high-risk regions that any additional impact of nosocomial transmission may remain modest. While antibiotic use was found to predispose to MDR colonisation as a whole, its association with contracting ESBL-PE was significant in univariate but not in multivariable analysis, possibly because of an insufficient number of observations.

The colonisation rates for other MDR bacteria were low, but not without relevance in a low-prevalence country like Finland [30]. In the stool specimens of 33 patients within this study, 46 carbapenemase-producing strains were recorded: 20 CPE and 26 MDRACI or MDRPA. In comparison, between 2010 and 2018, only 136 CPE strains were reported for the entire Helsinki and Uusimaa hospital district serving a population of 1.7 million, and the rates of MDRACI and MDRPA have been very low [30,31]. Thus, direct hospital transfers evidently contribute considerably to these cases. The MRSA colonisation rate of 3.6% is in line with that of 1.2–4.1% observed in earlier studies [5-8,12]. Although the rates for MRSA (3.6%) and VRE (3.8%) may appear low, they exceed those typical for Finland in general [30].

In univariate analyses conducted separately for MDRACI, MRSA and VRE, we found several associations. Antibiotic use abroad and ICU treatment were associated with each of the three. Indeed, both antibiotic use and intensive care predispose to MDR acquisition [24-26]. The association observed between MRSA carriage and chronic alcohol abuse confirms results of a previous study [32].

Clinical, microbiologically verified MDR bacterial infections were identified in 10.6% of the colonised patients in our study within 30 days after transfer, consistent with the rate of 11.4% observed in 2010 to 2013 as reported by Khawaja et al. [13] The rate of infection observed in the study by Mutters et al. was significantly higher at 29.9%, however, the demographics of patients in that study were different: over half of the patients were transferred to Germany from their country of origin in the Middle East [12]. As for non-hospitalised healthy travellers, we recently showed a maximum clinical infection rate of 17% for ESBL-PE carriers, with travellers’ diarrhoea (TD) as the most common manifestation, whereas the estimated maximal rate of infections other than TD was 3% [4]. Indeed, the data suggested higher rates of clinical MDR infections (TD excluded) among travellers who were hospitalised compared with non-hospitalised travellers [4].

### Limitations of the study

This investigation had limitations typical of a retrospective study design, such as incomplete data in some patient records and missing information on pre-travel colonisation. Comparisons can nevertheless be made with background colonisation rates, as mentioned above. Due to lack of non-hospitalised controls, it is difficult to determine the respective proportions of nosocomial and community-acquired infection during travel. In numerous reports on ESBL-PE colonisation after travel without hospitalisation [1] the rates resemble our data, but community acquisition of other MDR bacteria appears limited [27,29,33-35]. Furthermore, since the time frame for recording MDR bacteria was up to 1 month, nosocomial transmission after return to Finland cannot be ruled out. However, the risk was considered marginal on account of the low background prevalence: only 2% of *S. aureus* isolates are MRSA strains [30], and the background colonisation rate of ESBL-PE in Finland remains under 5% [28].

As culture-based assays lack sensitivity, a greater number of different MDR bacterial strains could be expected when employing modern genome-based methods, as shown in our recent study on travellers [36]. However, culture-based approaches are used in clinical practice and allow comparisons with earlier studies. For some patients, MDR bacteria from non-screened anatomic sites, such as skin lesions, could have been missed. However, we believe that the strict inclusion criteria for triple-site MRSA and faecal MDRGNB screenings together with clear hospital guidelines have resulted in a realistic yield.

With regards to antibiotic treatments, records from hospitals abroad were often not available, and antibiotics in use at the time of screening may have affected MDR bacterial findings. In general, depending on the setting, a concomitant antimicrobial effect may lead to unsuccessful bacterial culture or, contrarily, a selection of resistant strains. The complex effects of various antibiotics and their combinations could not be analysed.

Finally, the rate of symptomatic MDR bacterial infections may be an underestimate, since infections without microbiological verification were not covered. As infections treated abroad and those diagnosed after discharge (or 30 days) were not recorded, a different design would be needed to evaluate the total burden of MDR bacterial infections among this patient population.

## Conclusions

Colonisation by MDR bacteria is common among patients transferred from hospitals in high-prevalence countries. The most prevalent bacteria, ESBL-PE, are also frequently carried by non-hospitalised travellers. In addition, a substantial number of non-ESBL-PE strains, such as carbapenemase-producing bacteria, was detected. Among the variety of risk factors of MDR bacterial colonisation that were identified, geographical region of hospitalisation proved the strongest predictor of MDR findings. The study indicates that systematic screening of international transfer patients is warranted; our data serve as valuable background for devising infection control policies.

## Supplementary Data

179000 Supplement
