# Supplementary material for: Import of multidrug-resistant bacteria from abroad through interhospital transfers, Finland, 2010–2019
Source: Euro Surveill. 2021 Sep 30;26(39):2001360. doi: 10.2807/1560-7917.ES.2021.26.39.2001360 (PMC8485579; doi:10.2807/1560-7917.ES.2021.26.39.2001360)
Supplement: Supplement [file 20-01360_KANTELE_Supplement.pdf]

This supplementary material is hosted by *Eurosurveillance* as supporting information alongside the article "Import of multidrug-resistant bacteria from abroad through interhospital transfers, Finland, 2010 to 2019" on behalf of the authors who remain responsible for the accuracy and appropriateness of the content. The same standards for ethics, copyright, attributions and permissions as for the article apply. Supplements are not edited by Eurosurveillance and the journal is not responsible for the maintenance of any links or email addresses provided therein.

Supplementary Table S1. Helsinki University Hospital MDR bacteria screening guidelines[a]

Issue year	Foreign countries covered	No. and timing of samples	Sampling sites
2010	All outside the Nordics	3<br>(0, 7 and 14 days from transfer to HUH)	**MRSA**: nose, throat, groin/perineum; if needed: wounds, catheter insertion sites, urine from indwelling catheter<br>**MDRGNB**: stool/rectum and throat/trachea[b]; if needed: wounds, urine for culture from indwelling catheter<br>**VRE**: stool; if needed: wounds
2012	All outside the Nordics	2 or 3<br>(MRSA and faecal/rectal MDRGNB: 0, 7 and 8 days; others: 0 and 7 days from transfer to HUH)	**MRSA**: nose, throat, groin/perineum; if needed: wounds, catheter/drain insertion sites, urine from indwelling catheter (if in place for over 7 days)<br>**MDRGNB**: stool/rectum; if needed: trachea[b], wounds, urine for culture from indwelling catheter (if in place for over 7 days)<br>**VRE**: stool/rectum; if needed: wounds
2016	All	2 (on separate days)	**MRSA**: nose, throat, rectum; if needed: wounds, trachea[b], urine from indwelling catheter (if in place for over 7 days)<br>**MDRGNB**: rectum; if needed: wounds, trachea[b], urine from indwelling catheter (if in place for over 7 days)<br>**VRE**: rectum; if needed: wounds, trachea[b], urine from indwelling catheter (if in place for over 7 days)
2019[c]	All	2 (on separate days)	**MRSA**: nose, throat, rectum/perineum; **VRE**: only rectum<br>Otherwise same as 2016

HUH: Helsinki University Hospital; MDR: multidrug-resistant; MDRGNB: multidrug-resistant Gram-negative bacteria screening package; MRSA: methicillin-resistant *Staphylococcus aureus*; VRE: vancomycin-resistant *Enterococcus*

[a] Screening is recommended in case of hospitalisation abroad lasting over 24 hours and/or if a medical procedure has been performed. The table is a summary of HUH guidelines translated by the authors.

[b] Applies to intubated/tracheostomized patients only.

Supplementary Table S2. Countries of hospitalisation[a]

**Region/country**	**No. of patients**
Europe	524
Spain	146
Estonia	107
Greece	32
Germany	31
Russian Federation	28
Italy	25
France	22
Austria	17
Sweden	16
Portugal	13
Latvia	10
Hungary	8
Poland	8
UK	7
Switzerland	6
Croatia	5
Cyprus	5
Norway	5
Bulgaria	4
Malta	4
Netherlands	4
Belgium	3
others	18
Asia[b]	96
Thailand	57
China	10
Afghanistan	5
Indonesia	4
India	3
others	17
Northern Africa, Middle East[c]	38
Turkey	19
Morocco	4
Egypt	3
Israel	3
United Arab Emirates	3
others	6
Sub-Saharan Africa[d]	21
Somalia	4

South Africa	3
others	14
Latin America, Caribbean	10
Oceania	6
Australia	5
others	1
North America	3
USA	3

[a] Countries of hospitalisation for patients directly transferred from hospitals abroad to Helsinki University Hospital, Finland, 2010–19 (n=698). Countries with at least three patients per country are shown separately, the rest as “others”.

[b] Two patients were both hospitalised in two different South-East Asian countries.

[c] One patient was hospitalised in two different countries within Northern Africa and Middle East.

[d] Four patients were hospitalised in two different Sub-Saharan countries.

Supplementary Table S3. Colonisation by ESBL-PE and MDR bacteria by year[a]

Screening year	No. of patients per year, n	MDR bacteria positive, n (%)	OR (95% CI)[a]	p value[b]	EBSL-PE positive, n (%)	OR (95% CI)[a]	p value[b]
2010	18	6 (33.3)	1.1 (0.5-2.8)	0.765	5 (27.8)	1.2 (0.5-3.2)	0.649
2011	47	17 (36.2)	1.3 (0.7-2.3)	0.362	12 (25.5)	1.1 (0.6-2.1)	0.741
2012	64	19 (29.7)	1.0 (0.6-1.6)	0.900	12 (18.8)	0.7 (0.4-1.4)	0.336
2013	53	16 (30.2)	1.0 (0.6-1.7)	0.975	13 (24.5)	1.1 (0.6-1.9)	0.866
2014	77	21 (27.3)	0.9 (0.5-1.4)	0.539	17 (22.1)	0.9 (0.5-1.5)	0.745
2015	86	28 (32.6)	1.1 (0.7-1.7)	0.654	26 (30.2)	1.4 (0.9-2.2)	0.145
2016	96	26 (27.1)	0.9 (0.5-1.3)	0.475	22 (22.9)	1.0 (0.6-1.5)	0.873
2017	87	25 (28.7)	0.9 (0.6-1.5)	0.733	15 (17.2)	0.7 (0.4-1.2)	0.149
2018	100	28 (28.0)	0.9 (0.6-1.4)	0.600	22 (22.0)	0.9 (0.6-1.5)	0.702
2019	70	22 (31.4)	1.1 (0.6-1.7)	0.843	19 (27.1)	1.2 (0.7-2.0)	0.471

CI: confidence interval; ESBL-PE: extended-spectrum β-lactamase-producing *Enterobacteriaceae*; MDR: multidrug-resistant; OR: odds ratio

[a] Colonisation by any MDR bacteria and ESBL-PE per year among patients transferred directly from hospitals abroad to Helsinki University Hospital, Finland, 2010–19 (n=698).

[b] Deviation from the overall level was determined for each year in univariate analysis.

Supplementary Table S4. Clinical MDR bacterial infections.

**Pathogen**	**Infection sites**
MRSA	pneumonia
MRSA	(non-surgical) wound infection
MRSA	sepsis, VAP
MDR *A. baumannii*, ESBL *K. pneumoniae*	VAP
ESBL *E. coli*	UTI
MRSA	VAP
ESBL *K. pneumoniae*	VAP
ESBL *E. coli*	SSI
ESBL *E. coli*, MDR *A. baumannii*, MDR *P. aeruginosa*	VAP
ESBL *K. pneumoniae*	UTI
ESBL *E. coli*	UTI
ESBL *E. cloacae*, ESBL *K. pneumoniae*	SSI
ESBL *K. pneumoniae*	empyema
ESBL *E. coli*	UTI
ESBL *E. coli*	SSI
ESBL *E. cloacae*	UTI
MDR *P. aeruginosa*	VAP
CPE *K. pneumoniae,* MDR *A. baumannii*	VAP
CPE *K. pneumoniae*	meningitis
MDR *A. baumannii*	VAP
ESBL *E. coli*	UTI
ESBL *K. pneumoniae*	SSI
CPE *E. coli*, CPE *K. pneumoniae*, ESBL *E. coli*	SSI
MRSA	SSI

[a] Multidrug-resistant pathogens and sites of clinical infections diagnosed after direct transfer from abroad to Helsinki University Hospital, Finland, 2010–19 (n=22/698 patients)

CPE: carbapenemase-producing *Enterobacteriaceae*; ESBL: extended-spectrum beta-lactamase; ESBL *E. cloacae*: ESBL-producing *Enterobacter cloacae*; ESBL *E. coli*: ESBL-producing *Escherichia coli*; ESBL *K. pneumoniae*: ESBL-producing *Klebsiella pneumoniae*; MDR: multidrug-resistant; MDR *A. baumannii*: MDR *Acinetobacter baumannii*; MDR *P. aeruginosa*: MDR *Pseudomonas aeruginosa*; MRSA: methicillin-resistant *Staphylococcus aureus*; SSI: surgical site infection; UTI: urinary tract infection; VAP: ventilator-associated pneumonia; VRE: vancomycin-resistant *Enterococcus*
